# Spatial heterogeneity and differential treatment response of acute myeloid leukemia and relapsed/refractory extramedullary disease after allogeneic hematopoietic cell transplantation

**DOI:** 10.1177/20406207221115005

**Published:** 2022-08-23

**Authors:** Desiree Kunadt, Sylvia Herold, David Poitz, Lisa Wagenführ, Theresa Kretschmann, Katja Sockel, Leo Ruhnke, Stefan Brückner, Ulrich Sommer, Frieder Meier, Christoph Röllig, Malte von Bonin, Christian Thiede, Johannes Schetelig, Martin Bornhäuser, Friedrich Stölzel

**Affiliations:** Department of Internal Medicine I, University Hospital Carl Gustav Carus, Dresden University of Technology (TU Dresden), Fetscherstrasse 74, 01307 Dresden, Germany; Institute for Pathology, University Hospital Carl Gustav Carus Dresden, Dresden University of Technology (TU Dresden), Dresden, Germany; Institute for Clinical Chemistry, University Hospital Dresden, Dresden, Germany; Department for Internal Medicine I, University Hospital Carl Gustav Carus Dresden, Dresden University of Technology (TU Dresden), Dresden, Germany; Department for Internal Medicine I, University Hospital Carl Gustav Carus Dresden, Dresden University of Technology (TU Dresden), Dresden, Germany; Department for Internal Medicine I, University Hospital Carl Gustav Carus Dresden, Dresden University of Technology (TU Dresden), Dresden, Germany; Department for Internal Medicine I, University Hospital Carl Gustav Carus Dresden, Dresden University of Technology (TU Dresden), Dresden, Germany; Department for Internal Medicine I, University Hospital Carl Gustav Carus Dresden, Dresden University of Technology (TU Dresden), Dresden, Germany; Institute for Pathology, University Hospital Carl Gustav Carus Dresden, Dresden University of Technology (TU Dresden), Dresden, Germany; Institute for Pathology, University Hospital Carl Gustav Carus Dresden, Dresden University of Technology (TU Dresden), Dresden, Germany; Department for Internal Medicine I, University Hospital Carl Gustav Carus Dresden, Dresden University of Technology (TU Dresden), Dresden, Germany; Department for Internal Medicine I, University Hospital Carl Gustav Carus Dresden, Dresden University of Technology (TU Dresden), Dresden, Germany; Department for Internal Medicine I, University Hospital Carl Gustav Carus Dresden, Dresden University of Technology (TU Dresden), Dresden, Germany; Department for Internal Medicine I, University Hospital Carl Gustav Carus Dresden, Dresden University of Technology (TU Dresden), Dresden, Germany; DKMS Clinical Trials Unit, Dresden, Germany; Department for Internal Medicine I, University Hospital Carl Gustav Carus Dresden, Dresden University of Technology (TU Dresden), Dresden, Germany; National Center for Tumor Diseases (NCT) Dresden, Dresden, Germany; German Cancer Consortium (DKTK) Partner Site Dresden, Dresden, Germany; German Cancer Research Center (DKFZ), Heidelberg, Germany; Department for Internal Medicine I, University Hospital Carl Gustav Carus Dresden, Dresden University of Technology (TU Dresden), Dresden, Germany; German Cancer Consortium (DKTK) Partner Site Dresden, Dresden, Germany

**Keywords:** allogeneic stem cell transplantation, AML, clonal landscape, extramedullary

## Abstract

Although extramedullary manifestations (EMs) are frequent in patients with acute myeloid leukemia (AML), they are often not detected during clinical workup and neither imaging- nor molecularly based diagnostic strategies are established to reveal their existence. Still, the detection of EM is essential for therapeutic decision-making, as EM present with aggressive and resistant disease and since mutational profiling might render patients within a different risk category, requiring personalized therapeutic strategies. Here, we report the case of an AML patient presenting with AML bone marrow (BM) infiltration and molecularly distinct EM at time of diagnosis followed by multiple EM relapses while undergoing several intensive chemotherapies including allogeneic hematopoietic cell transplantations (alloHCTs). ^18^Fluorodesoxy-glucose positron emission tomography (^18^FDG-PET)-imaging revealed EM sites in the mediastinum, duodenum, skin, and in retroperitoneal tissue, whereas recurrent BM biopsies showed continuous cytomorphologic and cytogenetic remission after alloHCT. To investigate the molecular background of the aggressive character of extramedullary disease and its differential treatment response, we performed amplicon-based next generation sequencing. An exon 4 (c.497_498insGA) frameshift *RUNX1* mutation was exclusively found in all of the patient’s EM sites, but not in the BM or in peripheral blood samples at time of EM reoccurrence. In addition, we detected an exon 13 (c.3306G>T) *ASXL1* point mutation only in the retroperitoneal tumor tissue at the time of the fourth relapse. In contrast to the patient’s intermediate-risk BM AML at diagnosis according to ELN2017, EM sites showed molecular adverse-risk features implicating intensified strategies like cellular therapies. Notably, disease relapse could only be detected by imaging throughout the course of disease. This case demonstrates both the necessity of continuous molecular profiling of EM to reveal differential molecular composition of EM and BM-derived AML, supposedly leading to divergent susceptibility to established therapies, as well as recurrent ^18^FDG-PET-imaging for detecting residual disease and assessment of treatment response in case of EM AML.

## Case presentation

**Figure 1.**
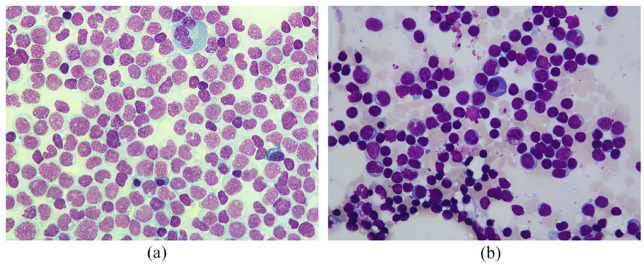
Cytomorphology of bone marrow aspirates. (a) Bone marrow aspirate smear at time of diagnosis (50× magnification) showed subtotal infiltration of myeloid blasts replacing normal hematopoiesis without evidence of myeloid maturation, leading to diagnosis of acute myeloblastic leukemia without maturation according to WHO criteria. (b) Bone marrow aspirate smear at time of relapse (50× magnification) revealed persistence of cytomorphologic remission with a blast count below 5%, myeloid maturation, and normal findings for erythroid and megakaryocytic components. Bone marrow smears (a and b) were stained with the May–Grunwald–Giemsa kit and examined with the Nikon Eclipse E600 microscope. High-resolution pictures were taken with the mounted Nikon DSFi2 camera and processed with the Nikon Imaging Software Elements.

A 25-year-old man presented with fatigue, recurrent episodes of fever and infectious symptoms, night sweats, and inappetence. In addition, dyspnea and chest pain during inspiration were reported. Furthermore, rash on both arms was described. Hematologic workup revealed leukocytosis of undifferentiated cells, which were characterized as myeloid progenitors by immunophenotyping. Bone marrow (BM) aspiration and biopsy confirmed the diagnosis of acute myeloid leukemia (AML) (Figure 1(a)). Cytogenetic analysis revealed an *NF1*-deletion (del17q11). No other genetic abnormalities were detected using routine molecular analysis methods [fragment length analysis and amplicon-based next generation sequencing (NGS)].

**Figure 2.**
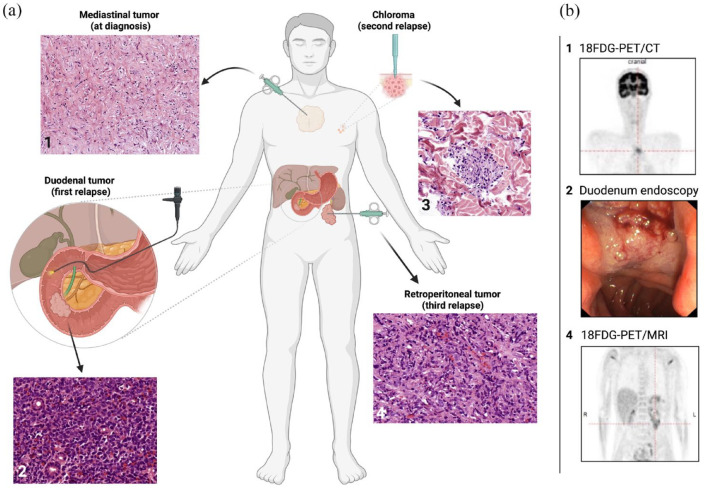
Overview of the patient’s extramedullary AML sites. The patient suffered from multiple extramedullary relapses of different sites (1, mediastinal tumor; 2, duodenal tumor; 3, chloroma; 4, retroperitoneal tumor). The chronology of the occurrence of the EM was as follows: first diagnosis with mediastinal tumor (1), first relapse with duodenal tumor (2), second relapse with chloroma (3), and third relapse with retroperitoneal tumor (4). All EMs were (a) histologically confirmed after EM tissue was obtained by CT-supported biopsy (1 and 4), by duodenoscopy (2), or by punch biopsy (3). Histologic samples were prepared with hematoxylin–eosin staining. Images were captured with the Olympus BX 41TF microscope (magnification 1: 18.7×, 2: 40×, 3: 40×, and 4: 40×) and the 3DHistech Pannoramic Desk. (b) 18FDG-PET/CT imaging revealed mediastinal EM manifestation at diagnosis (b1), duodenoscopy pictured the duodenal tumor (b2), and follow-up 18FDG-PET/MRI depicted the retroperitoneal manifestation (b4). Figure was created with BioRender.

Induction chemotherapy with 60 mg/m^2^ daunorubicin (day 3–5) and 100 mg/m^2^ cytarabine (day 1–7) (DA) was initiated. As the patient complained of chest pain and newly onset of fever episodes, computed tomography (CT) of the thorax was performed on day 6 of DA, depicting a large mediastinal tumor and ground-glass opacities. ^18^Fluorodesoxy-glucose positron emission tomography/computed tomography (^18^FDG-PET/CT) demonstrated mediastinal vital tissue [maximum standardized uptake value (SUV_max_) 5.3; Figure 2(b1)]. Hence, a biopsy of the tumor was conducted, revealing residual leukemic blasts embedded in necrotic tissue through histologic workup, being the first verified extramedullary site of the patient’s AML (Figure 2(a1)).

BM assessment at day 15 showed moderate response and positive cytogenetic measurable residual disease (MRD^pos^) with persisting del17q11 detection. A second cycle of DA was initiated leading to cytomorphologic and cytogenetic complete remission (CR). Follow-up CT showed the mediastinal tumor unchanged in diameter. No further extramedullary manifestation (EM) sites were traced in clinical examination and by imaging.

The patient underwent allogeneic hematopoietic cell transplantation (alloHCT) from a matched sibling donor after receiving myeloablative conditioning with 120 mg/kg cyclophosphamide and 12 Gy fractionated total body irradiation (TBI), which was tolerated without unexpected toxicities. Graft *versus* host disease (GvHD) prophylaxis consisted of methotrexate (MTX) and cyclosporin (CsA). On day +8, the patient reported sudden and severe chest pain again and imaging revealed the mediastinal tumor with unchanged diameters. The patient developed full donor chimerism until day +56 and remained in cytomorphologic and cytogenetic CR. Immunosuppression was tapered rapidly and discontinued 6 months after transplantation.

Eighteen months later, the patient noticed progressive jaundice. Abdominal ultrasound revealed a large tumor of the pancreaticoduodenal region causing cholestasis. Endoscopy revealed a tumor of the duodenum obstructing the common hepatic duct with the need of stent insertion (Figure 2(b2)). Biopsies were obtained, again demonstrating leukemic infiltration and defining AML relapse (Figure 2(a2)). Cytogenetic evaluation of the duodenal tumor was indicative for the known *NF1*-deletion. In addition, ^18^FDG-PET/magnetic resonance imaging (MRI) was further suspicious for localized iliac EM (SUV_max_ 5.4), while the initial mediastinal tumor (SUV_max_ 3.5–5.2) and the duodenal tumor (SUV_max_ 3.7) also showed metabolic activity. Surprisingly, BM aspiration showed a persisting CR (Figure 1(b)) without cytogenetic or molecular abnormalities and full donor chimerism. Reinduction with 3000 mg/m^2^ BID high-dose cytarabine (day 1–3) and 5 mg/m^2^ mitoxantrone (day 3–5, dose reduction because of pancreatitis) was started, which induced a metabolic CR of the EM according to ^18^FDG-PET/MRI. At the time of referral to the transplantation ward for second alloHCT, the patient reported about a cutaneous tumor on the chest that rapidly grew within the last few days. A punch biopsy verified again leukemic infiltration in this lesion (Figure 2(a3)). Myeloablative conditioning with 12.8 mg/kg busulfan and 150 mg/m^2^ fludarabine was applied prior to allografting from an alternative matched unrelated donor (MUD). MTX and CsA were applied as GvHD prophylaxis. Full donor chimerism was achieved again; no further sites of chloroma appeared. BM aspirate revealed persisting CR. The patient developed acute GvHD grade III of the upper and lower gastrointestinal tract and the skin, which required repetitive escalation of immunosuppression. However, a follow-up ^18^FDG-PET/MRI demonstrated relapse of the mediastinal mass 1 month after the second transplantation (SUV_max_ 6.6). Local radiation therapy of the mediastinal tumor with a cumulative dose of 40 Gy was conducted leading to a significant shrinking of the mediastinal tumor burden. Three months later, the patient showed new signs of progression, this time with multiple EMs detected below the left kidney causing urinary obstruction, paravertebrally, and in the coeliac trunk (SUV_max_ 4, Figure 2(b4)). Again, myelosarcoma was histologically confirmed after biopsy (Figure 2(a4)). Therapy with azacitidine and venetoclax was initiated, resulting in a morphologically complete response after two cycles of chemotherapy as per ^18^FDG-PET/MRI. To study the clonal landscape of this aggressive and refractory course of extramedullary disease, we performed amplicon-based NGS of AML tumor tissue and compared it with genetic characteristics of the patient’s BM AML.

## Methods

Genomic DNA was extracted from pre-treatment and relapse BM aspirates and EM sites obtained at time of occurrence. For BM samples, the Maxwell^®^ RSC Buffy Coat DNA Kit (Promega, Madison, WI, USA) was used; for formalin-fixed, paraffin-embedded (FFPE) EM material, the QIAamp^®^ DNA Mini Kit (Qiagen^®^, Hilden, Germany) was used. In the case of FFPE material, tumor area was marked before on a hematoxylin–eosin (HE) stained slide, and only tumor areas were utilized for DNA extraction. DNA concentration was quantified using the QuantiFluor^®^ dsDNA System (Promega) in case of BM samples and the Qubit™ dsDNA BR Assay (Thermo Fisher Scientific, MA, USA) for DNA isolated from FFPE samples. In addition, DNA extracted from a salivary sample obtained at diagnosis with the ORAgene One kit (DNAgenotek^®^, Ottawa, ON, Canada) was included as germline control.

In total, 100 ng of DNA was used as input for library preparation. The regions of interest were amplified using a custom designed amplicon NGS panel (QIAseq Targeted DNA V3 Panel, May 2017; Qiagen^®^), which was designed particularly for AML. The panel covers mutation hotspots or whole genes of *ASXL1*, *BRAF*, *CBL*, *CEBPA*, *DNMT3A*, *FLT3*, *GATA2*, *IDH1*, *IDH2*, *JAK2*, *KRAS*, *MPL*, *NPM1*, *NRAS*, *RUNX1*, *SETBP1*, *SRSF2*, *TET2*, *TP53*, *U2AF1*, *WT1*, and *ZRSR2*. During library preparation, unique molecular barcodes and sample-specific indices were incorporated according to the manufacturer’s protocol. Indexed libraries were then quantified using a Qubit dsDNA HS Assay Kit (Thermo Fisher Scientific) and paired-end sequenced (2 × 200 bp) on an Illumina^®^ MiSeq platform (San Diego, CA, USA). HG19 was used as reference genome for bioinformatic analyses. The bioinformatics evaluation was performed using the Biomedical Workbench from CLC (version 21.0.3, Qiagen^®^) using a customized analysis algorithm with the following filters: coverage ⩾200 and allele frequency ⩾1%. Synonymous sequence variants and polymorphisms were not reported. Sequence variants with a change in the amino acid sequence were specified according to HGVSc and HGVSp with details of the underlying reference sequence and allele frequency. NGS-based assessment of MRD for single nucleotide variants (SNVs) was performed using an error-controlled deep sequencing procedure, as described in detail recently.^1^ The study was reported according to the CARE guideline (Supplementary File).^2^ Patient details were de-identified.

## Results

**Figure 3.**
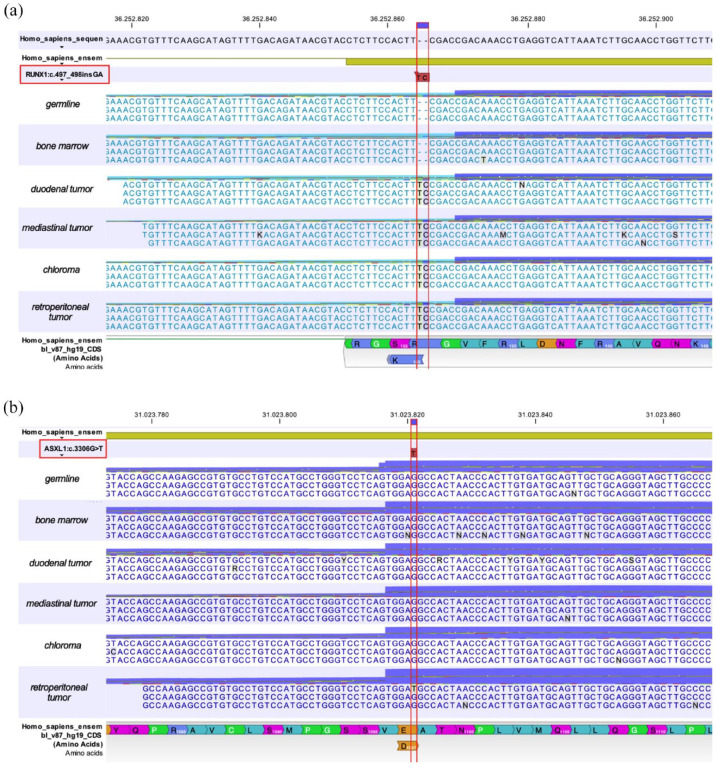
Myeloid panel sequencing of EM, germline, and bone marrow samples. (a) Myeloid panel sequencing revealed recurrent *RUNX1*:c.497_498insGA frameshift mutation in exon 4 in all extramedullary sites, but not in the bone marrow aspirate or in germline DNA. (b) *ASXL1*:c.3306G>T point mutation in exon 13 was only detected in the retroperitoneal and latest EM site, while bone marrow, germline DNA, and other EM of the patient showed no *ASXL1* aberration at previous AML disease stages.

Except for an *NF1*-deletion (del17q11) revealed by cytogenetic analysis, no AML-specific genetic abnormalities according to the ELN2017 classification^3^ were found in the BM at diagnosis, classifying the patient’s AML as intermediate-risk. An *RUNX1* frameshift mutation in exon 4 (c.497_498insGA) with a variant allele frequency (VAF) of 31.1% could be detected in the mediastinal EM from initial diagnosis (Figure 3(a)), however, suggesting the existence of two molecularly and anatomically distinct AML clones at time of diagnosis as the *RUNX1* mutation was not detected in the BM. First relapse was characterized by a duodenal EM, while BM cytomorphology stated continuous CR (Figure 1(b)) without any former or new molecular and cytogenetic aberrations. Also, NGS-based MRD analysis of peripheral blood (pB) samples at time of first relapse did not reveal the presence of the known *RUNX1* exon 4 (c.497_498insGA) mutation (limit of detection 0.1%). In contrast, the duodenal AML manifestation harbored the same exon 4 *RUNX1* mutation (VAF 26%) as the initial mediastinal manifestation (Figure 3(a)). Mutational analysis of EM tissue from the second relapse also demonstrated the previously detected exon 4 *RUNX1* mutation (VAF 18.9%) (Figure 3(a)). All three EM sites did not harbor other AML-specific molecular aberrations as analyzed by the custom amplicon panel. To rule out germline involvement, salivary DNA obtained at diagnosis was also investigated by NGS and demonstrated no molecular markers. As AML progressed again with the fourth EM site affecting perirenal, paravertebral, and perivascular tissue, we continued molecular monitoring of EM. This time, along with the known *RUNX1* mutation (VAF 40.6%) (Figure 3(a)), we further detected an exon 13 (c.3306G>T) *ASXL1* point mutation with a VAF of 6.3% in the respective EM tissue (Figure 3(b)). Again, the *RUNX1* mutation could not be detected in a pB sample. Noteworthy, recurrent BM examinations stated continuous CR with molecular MRD-negativity (MRD^neg^) after achieving CR and MRD^neg^ after first alloHCT. Furthermore, pB and BM samples demonstrated full donor chimerism throughout the following course of disease, making it impossible to detect AML reoccurrence *via* routine laboratory tests.

## Discussion

Although the prognosis for patients with AML has improved significantly over the last decades, EMs still represent a clinical dilemma because of a variety of affected tissues, highly resistant disease characteristics, and frequent relapse even in the face of intensive treatments.^4–7^ The prospective PETAML trial demonstrated EM occurring frequently at time of diagnosis (22%) with the majority of these AML patients still harboring active EM in follow-up imaging after chemotherapy despite CR in BM.^6^ Still, EMs are often under-reported during routine clinical workup, as some manifestations are only detectable by in-depth imaging series revealing only the ‘tip of the iceberg’. Interestingly, EM can occur and relapse with or without concomitant BM infiltration, suggesting differential disease biology.^8,9^ There have been a few reports demonstrating differences in metabolic pathways and forms of immune escape phenomena between BM AML and EM AML.^10,11^ Also, the clonal composition of EM in contrast to BM-derived AML as well as unique features and differences in genetic and phenotypic characteristics causing EM disease biology have not been investigated comprehensively. There is emerging evidence on significant differences and substantial heterogeneity in clonal composition of EM *versus* BM AML, however, begging the question of a (simultaneous) development of distinct AML clones and sites with different genetic features, disease characteristics and kinetics, as well as vulnerable targets to elaborate that might translate into adjusted therapeutic requirements.^12–16^

Here, we report a paradigmatic case of a young patient with two distinct AML clones at diagnosis, which differed both in genetic markers, disease localization, as well as response to leukemic treatment and disease kinetics. In contrast to routine hematologic and genetic workup of the BM at diagnosis classifying the patient’s AML as intermediate-risk according to the ELN2017 classification, EM sites showed genetic adverse-risk features (Supplementary Table 1) implicating worse prognosis and the need for intensified strategies like cell-based approaches. We assume that these differences of BM AML and EM AML are one of the components for differential response and the aggressive character of many EMs. We further hypothesize that the differential features of EM AML compared with BM AML may possibly be a general underlying concept, especially in AML cases with refractory behavior, aggravated by the ability of EM sites to undergo clonal evolution and intensify immune escape. The escape of EM at ‘sanctuary sites’ is also reflected by the frequent progression of EM after alloHCT despite the occurrence of clinically significant acute and chronic GvHD, as seen in our patient.^9,17^ As the clinical course described herein suggests, EM may undergo clonal evolution throughout sequential treatment by gaining additional mutations conveying resistance against the chosen salvage regimens. Further studies should focus on the clonal heterogeneity of extramedullary disease, the differences in phenotypic properties and genetic composition compared with their BM counterparts, and potential changes during the course of disease.

In our case, rapid diagnosis of disease reoccurrence was complicated by the fact that EM progression could not be detected with routine donor chimerism analyses, as full donor chimerism was present throughout the course of disease, or by NGS analyses of pB samples. Therefore, imaging remains an essential component for the detection of recurrent disease in patients with EM.

As this case illustrates, improved and targeted therapies are needed for EM AML patients. Recently, novel *RUNX1*-targeted approaches showed promising results in a murine AML model and may pave the way to future opportunities to address refractory EM based on genetic data.^18^ Furthermore, the immune checkpoint inhibitor ipilimumab was able to induce complete responses in single cases of relapsed AML patients with EM after alloHCT, making it an attractive molecule for further investigations.^19^

Another challenging aspect of EM AML is its potential to invade or to arise in various tissues, respectively, whereas it is still unclear whether this distribution also relies on specific features or happens by coincidence. The PETAML trial determined a median number of two EM sites per patient (range 1–12), a finding that was also confirmed in our patient with both skin and mediastinal involvement at diagnosis. A lesson learnt from this case vignette is the added information that may be derived from repetitive biopsies with histological and molecular workup. Also, repeated ^18^FDG-PET imaging is necessary for both revealing EM sites suitable for diagnostic puncture, as well as the detection of disease reoccurrence and response assessment.

Importantly, treatment of AML patients with EM is an interdisciplinary effort, both in case of organ involvement but also during acquisition of biopsy material and diagnostic workup. Further research is needed to address drivers and pathways of clonal evolution, metabolic components, as well as characteristic traits of differential EM sites. Results may refine our concepts of EM and improve miscellaneous therapeutic strategies and future targeted approaches beyond standard AML therapies.

## Supplemental Material

sj-docx-1-tah-10.1177_20406207221115005 – Supplemental material for Spatial heterogeneity and differential treatment response of acute myeloid leukemia and relapsed/refractory extramedullary disease after allogeneic hematopoietic cell transplantation Click here for additional data file. Supplemental material, sj-docx-1-tah-10.1177_20406207221115005 for Spatial heterogeneity and differential treatment response of acute myeloid leukemia and relapsed/refractory extramedullary disease after allogeneic hematopoietic cell transplantation by Desiree Kunadt, Sylvia Herold, David Poitz, Lisa Wagenführ, Theresa Kretschmann, Katja Sockel, Leo Ruhnke, Stefan Brückner, Ulrich Sommer, Frieder Meier, Christoph Röllig, Malte von Bonin, Christian Thiede, Johannes Schetelig, Martin Bornhäuser and Friedrich Stölzel in Therapeutic Advances in Hematology

## Declarations

**Ethics approval and consent to participate:** The patient was enrolled in the SAL registry (NCT03188874) at diagnosis including his informed consent for routine sample collection to be stored as samples for scientific research afterwards, data collection, and analysis of data for publication. The SAL registry was approved by the respective ethics committee of the TU Dresden, Germany (EK98032010) and is conducted in accordance with the Declaration of Helsinki.

**Consent for publication:** All authors read the manuscript and gave their final approval for publication.

**Availability of data and materials:** The datasets used and/or analyzed during the current study are available from the corresponding author on reasonable request.
