# Supplementary material for: Spatial heterogeneity and differential treatment response of acute myeloid leukemia and relapsed/refractory extramedullary disease after allogeneic hematopoietic cell transplantation
Source: Ther Adv Hematol. 2022 Aug 23;13:20406207221115005. doi: 10.1177/20406207221115005 (PMC9425876; doi:10.1177/20406207221115005)
Supplement: sj-docx-1-tah-10.1177_20406207221115005 – Supplemental material for Spatial heterogeneity and differential treatment response of acute myeloid leukemia and relapsed/refractory extramedullary disease after allogeneic hematopoietic cell transplantation [file sj-docx-1-tah-10.1177_20406207221115005.docx]

**Supplement**

**Supplemental Table 01**

|  | **First Diagnosis** | | **1st relapse** | **2nd relapse** | **3rd relapse** |
| --- | --- | --- | --- | --- | --- |
| **Genetic aberration** | **Bone Marrow** | **Mediastinal tumor** | **Duodenal tumor** | **Chloroma** | **Retroperitoneal tumor** |
| ***NF1-*deletion** | 69 % | *unknown* | 38 % | *unkown* | *unknown* |
| ***RUNX1*** | negative | VAF 31.1 % | VAF 26 % | VAF 18.9 % | VAF 40.6 % |
| ***ASXL1*** | negative | negative | negative | negative | VAF 6.3 % |
| **ELN2017** | intermediate | adverse | adverse | adverse | adverse |

**Suppl. Table 01 Overview of the patient’s genetic aberrations**

Both the cytogenetic (*NF1* deletion) and molecular findings (*RUNX1* and *ASXL1* mutation) are depicted for the different extramedullary sites and the bone marrow. VAF, variant allele frequency.
